# Defining the Tumor-Bone axis: the role of extracellular vesicles in a crucial exchange route for breast cancer development and progression

**DOI:** 10.1007/s10585-025-10377-8

**Published:** 2025-10-17

**Authors:** Carlos D. Coronado-Alvarado, Humberto Astiazaran-Garcia

**Affiliations:** 1 Centro de Investigación en Alimentación y Desarrollo, A. C. Carretera Gustavo Enrique Astiazarán Rosas, No. 46, Col. La Victoria, CP. 83304 Hermosillo, SON México; 2 Departamento de Ciencias Químico-Biológicas, Universidad de Sonora, Blvd. Luis Encinas y Rosales S/N, Centro, 83000 Hermosillo, Sonora México

**Keywords:** Breast neoplasms, Bone remodeling, Hematopoiesis, Extracellular vesicles, Tumor microenvironment

## Abstract

Bone disorders frequently manifest as long-term outcomes of breast cancer. Consequently, the relationship between breast cancer and bone metabolism is often studied at advanced stages of the disease. Emerging evidence suggests that bidirectional communication between mammary and bone tissues begins much earlier. In this context, extracellular vesicles (EVs) have been recognized as key mediators of intercellular communication, with emerging evidence supporting their role in breast cancer progression and the regulation of bone metabolism. This review examines bone imbalances occurring throughout the course of breast cancer, the pathophysiological mechanisms behind them, and the role of EVs in their development. From this integrated perspective, we propose the concept of Tumor-Bone Axis, a continuous and dynamic crosstalk between breast cancer and bone cells that supports tumor progression and bone complications. This axis regulates distinct metabolic states governing the activity of breast cancer cells and the balance in bone remodeling, enabling cellular reprogramming events during malignant transformation, immunoediting, tumor growth, and metastasis formation. Additionally, the impact of antineoplastic treatments on this axis may underlie chemoresistance, relapse, or therapy-induced metastasis. While multiple mediators are involved—including cell-to-cell contact, cell migration, osteoimmune interactions, hormones, soluble factors, and nutrients—EVs appear to be critical, especially through their role in exchanging epigenetic regulators of central signaling pathways in these cellular reprogramming events. Understanding the temporal and functional dynamics of the Tumor-Bone Axis and the extracellular vesicular traffic within it could reveal novel diagnostic biomarkers and therapeutic strategies for both breast cancer and its bone-related manifestations.

## Introduction

Significant efforts have been made to develop therapeutic strategies against breast cancer, currently the most prevalent malignancy worldwide [1]. While these advancements have allowed for improved survival, long-term complications and recurrences of the disease remain major challenges. Among them, bone disorders in breast cancer (BDBC) are common, ranging from often-overlooked metabolic bone changes to serious skeletal-related events (SREs), such as bone pain, fragility fractures, and calcium imbalances [2]. SREs typically result from either bone metastases or cancer treatment-induced bone loss (CTIBL), both late-stage and difficult-to-predict processes [2]. Consequently, BDBC are usually approached as long-term outcomes subject to the same constraints as these two processes, and thus, they might benefit from a different analytical framework.

In past decades, several hypotheses have challenged oncological paradigms, enhancing our understanding of cancer development and progression [3–11]. These include the wound-oncogene-wound healing theory, cancer stem cells, immunoediting, systemic evolutionary theory of cancer, metastasis patterns, and the vicious cycle of bone metastasis, among others. While each focuses on a specific aspect of cancer pathophysiology, a holistic view—explicitly applied to BDBC—may reveal new insight and opportunities for research. A shared premise of these theories is the reconceptualization of cancer from a cell-originated disease to a tissue-level condition driven by dysregulated intercellular communication [6], sometimes involving distant tissues, such as the breast and bone, as evidenced by metastasis. Therefore, characterizing these altered communications could be a foundational step in studying BDBC.

Recently, extracellular vesicles (EVs) have emerged as key mediators of intercellular communication. These heterogeneous, cell-released, membranous nanoparticles can carry diverse molecular cargoes and are detectable in various tissues and biofluids [12]. By protecting their contents from degradation, EVs enable the targeted exchange of molecules between cells, facilitating communication across short and long distances without relying on external regulatory systems such as the nervous, endocrine, or immune systems [12]. In bone metabolism, EVs initiate calcification and coordinate the activities of multiple resident cell types [13]. Additionally, they participate in critical oncological processes, including tumorigenesis, immunoediting, and tissue invasion [14, 15]. Growing evidence also implicates EVs in the crosstalk between tumor and bone cells in breast cancer, particularly in osteomimicry, premetastatic niche formation, and the persistence of disseminated tumor cells within bone tissue [16]. As such, they could be involved in the development of BDBC.

In this work, we examine, through the lens of current oncological theories, the alterations in bone metabolism that occur in breast cancer and explore the potential role of EVs in these processes. Based on this integrative analysis, we propose the Tumor-Bone Axis, a theoretical framework emphasizing the sustained and dynamic crosstalk between mammary and bone cells as a critical factor in breast cancer progression and the onset of BDBC. This framework incorporates both emerging and established concepts—such as osteoimmune interactions, immunoediting, breast osteoblast-like cells, or the pre-metastatic niche—by interpreting them as a continuum, rather than isolated phenomena, as they are typically studied. Framing these elements within the context of BDBC offers new perspectives, revealing distinct metabolic environments that shape their progression, with EVs consistently implicated as a key mediator in their development. Finally, we identify key knowledge gaps and discuss the properties that EV cargo may require to drive these different metabolic environments.

## Bone metabolism changes in breast cancer

Bone metabolism involves a complex set of highly active, lifelong, and coordinated processes. Clinically, the two most important are hematopoiesis and bone remodeling. Hematopoiesis refers to the differentiation of hematopoietic stem cells (HSCs) into various cell types, including immune cells and platelets [17]. Bone remodeling encompasses the coupled activities of bone resorption and bone formation, executed respectively by osteoclasts and osteoblasts [18]. Following bone formation, osteoblasts may undergo apoptosis or differentiate into osteolineage cells, becoming either osteocytes embedded in the bone matrix or bone-lining cells covering the endosteal surfaces [19]. Osteocytes, the most abundant bone cells, possess mechanosensitive properties and regulate bone remodeling by releasing modulators of osteoclasts and osteoblasts activity, such as RANKL or sclerostin, respectively [13, 19].

**Fig. 1:**
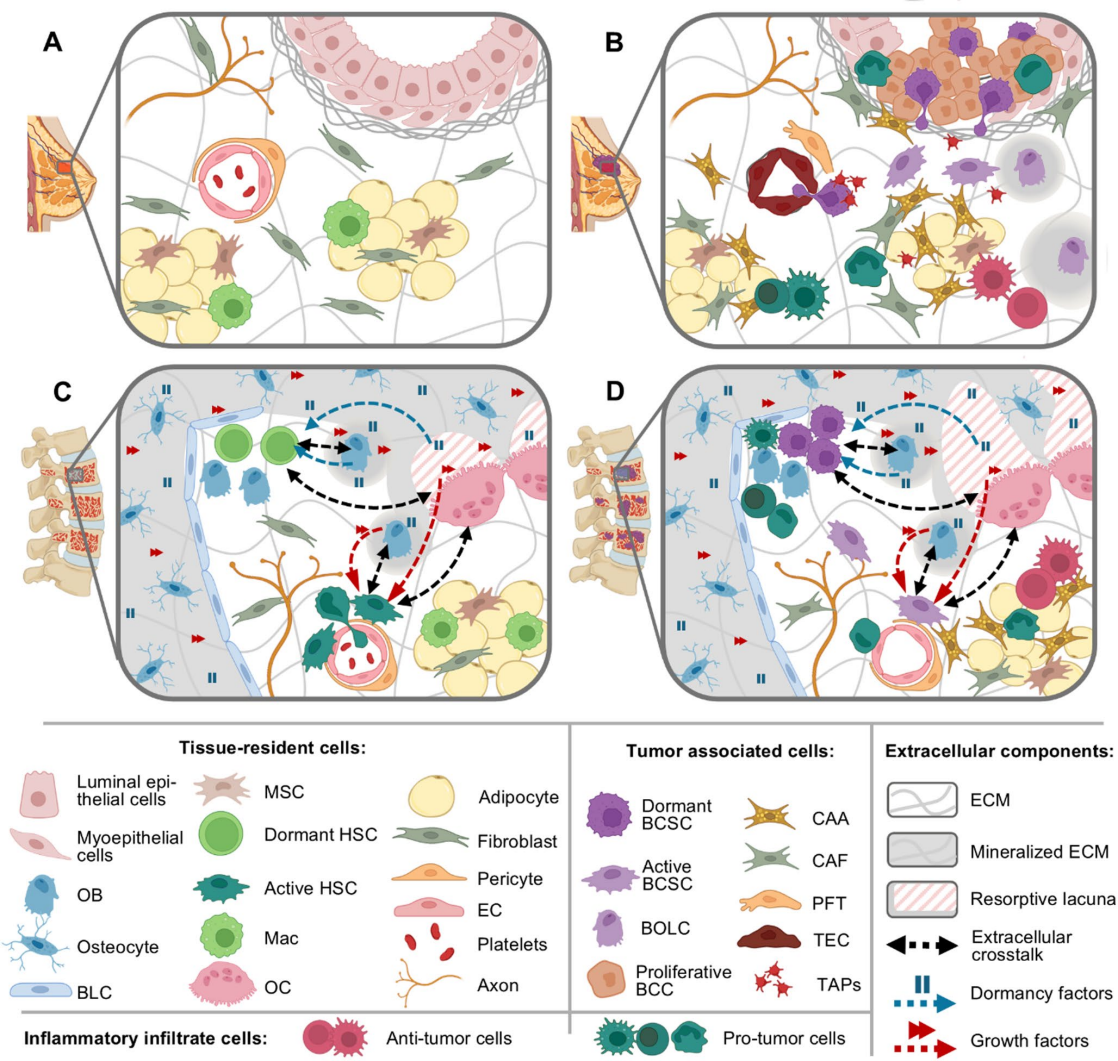
Cellular elements in the different microenvironments comprising the Tumor-Bone Axis. (**A**) Several cells reside in normal breast tissue. (**B**) During tumorigenesis, these cells undergo metabolic reprogramming and phenotypic transformation, developing the primary TME, composed of tumor-associated cells and both pro-tumor and anti-tumor inflammatory infiltrates. BCSCs can generate proliferative BCCs or undergo EMT, acquiring osteomimetic traits and increased invasiveness. (**C**) During normal bone metabolism, HSCs in endosteal niches (surrounded by OBs) are typically dormant, whereas those in perivascular niches are usually active. These cells engage in modulatory crosstalk with OBs and OCs, which produces dormancy- and growth-related factors that regulates hematopoiesis. Some of these factors become embedded in the bone matrix during ossification and release during resorption. (**D**) In invaded bones, metastatic cells occupy the HSC niches, induce metabolic alterations in both OBs and OCs, hijack the dormancy and growth signals originally intended for HSCs, and reprogram local stromal cells into tumor-associated cells. BCC, Breast cancer cell; BCSC, breast cancer stem cell; BLC, bone lining cell; BOLC, breast osteoblast-like cell; CAA, cancer-associated adipocyte; CAF, cancer-associated fibroblast; EC, endothelial cell; ECM, extracellular matrix; EMT, epithelial-mesenchymal transition; HSC, hematopoietic stem cell; Mac, macrophage; MSC, mesenchymal stem cell; PFT, pericyte-to-fibroblast transitioning cell; OB, osteoblast; OC, osteoclast; TAPs, tumor-associated platelets; TEC, tumor-associated endothelial cells; TME, tumor microenvironment

Notably, other bone-resident cells can also be normally found in breast tissue, including mesenchymal stem cells (MSCs), adipocytes, fibroblasts, endothelial cells, pericytes, Schwann cells, and autonomic axons (Fig. 1). All bone cells are organized into specialized bone marrow niches, each with distinct regulatory functions essential for bone remodeling and hematopoiesis [20–22]. Disruptions in these cellular interactions and organization lead to metabolic disturbances. Conditions such as nutritional deficiencies, autoimmune or endocrine disorders, chronic kidney disease, and malignancies—including breast cancer— may cause such disruptions, leading to failures in both bone remodeling and hematopoiesis [23–29].

The interplay between bone marrow niches—and thus between hematopoiesis and bone remodeling—is essential for maintaining bone homeostasis. Osteoblasts originate from MSCs, whereas osteoclasts derive from HSCs, making both processes intrinsically interconnected [30]. Immune cells derived from HSCs can shape the bone environment into either an inflammatory osteolytic or an anti-inflammatory osteogenic state during bone remodeling [30], an aspect particularly relevant in BDBC. Conversely, bone remodeling cells secrete growth and dormancy factors that regulate HSC metabolism. These factors can become embedded in the bone matrix during ossification and later released through resorption, thereby influencing HSC maintenance [31]. Furthermore, osteolineage cells are critical to hematopoiesis, contributing to the extracellular matrix (ECM) synthesis and supporting HSC mobilization across niches [17, 32]. HSC localization is tightly linked to their stemness—that is, their capacity for self-renewal, quiescence, and differentiation. Although some evidence challenges current models of bone marrow organization [33, 34], proliferating HSCs are typically found in perivascular niches, while dormant ones are found in endosteal niches, surrounded by osteolineage cells [22] (Fig. 1C).

Communication between hematopoietic cells and neighboring bone cells is bidirectional and mediated by numerous signaling molecules. Hematopoietic cells produce factors such as RANK-L, IgG, IL-4, IL-10, IL-13, IL-17, TNF, and IL-6, which promote osteoclast activity; IFNγ, which inhibits it; IL-17 and TGF-β, which enhance osteogenic activity; and G-CSF, which suppresses it [35–43]. In turn, osteolineage cells regulate HSCs through factors such as RANK-L, CXCL12, DLL4, IL-7, DKK1, and EPO [42–47]. Osteoclasts influence hematopoiesis through matrix proteases, Cathepsin K, chemokines, and bone-derived factors released during osteolysis [41, 48–54]. Other organs also modulate this interplay. For instance, metabolites from the intestinal microbiota and signals from tumors—sometimes even in the absence of bone metastases—can affect bone remodeling and hematopoiesis [55–58]. Notably, EVs may serve as key intermediaries in these processes [59]. While many of these osteoimmune interactions are increasingly understood—particularly in autoimmune, infectious, and hematologic diseases [41]—further research is needed to elucidate their precise mechanisms in the context of BDBCs.

In breast cancer, both hematopoiesis and bone remodeling can be disrupted by various factors. As an inflammatory condition, breast cancer is associated with elevated cytokine levels that promote hematopoietic alterations—primarily a shift toward myeloid lineage expansion. This is reflected by clinical findings such as leukocytosis, anemia, increased red cell distribution width, clonal hematopoiesis of indeterminate potential, and, less frequently, extramedullary hematopoiesis [60–65]. Although chemotoxicity from antineoplastic treatments significantly contributes to these disturbances, some alterations appear prior to therapy, suggesting a direct link between tumor development and hematopoietic dysfunction [62, 65], These alterations may reflect complex pathophysiological processes involving hematopoiesis-derived cells that contribute to tumor progression and premetastatic niche formation, influencing bone remodeling [66–70].

Preclinical models have shed light on the cooperative interaction between bone cells and breast cancer cells, particularly in the context of metastasis. During lung metastasis, primary breast tumors can secrete hypoxia-associated factors to recruit hematopoietic-derived cells, including myeloid-derived suppressor cells (MDSCs), to the premetastatic site [71–73], facilitating immune evasion and ECM remodeling [67, 74]. In bone metastasis, premetastatic niche formation involves changes in ECM composition, nutrient availability [75, 76], and osteoblast-induced release of collagen fragments that attract tumor cells [77]. A reduction in MSCs—especially perivascular ones and those supporting myelopoiesis—has also been observed [78, 79]. Tumor-derived factors favor osteoclast activity over osteogenesis, partially mediated by immune cells such as CD4+/CD8 + T cells, B cells, and dendritic cells [68–70, 80–83]. These findings support the idea that secondary tumors in bone require an osteolytic environment, consistent with the “vicious cycle” model of bone metastases, in which tumor cells drive bone resorption to release osteolysis-derived factors (such as TGF-β, Calcium, or IGF-1) that enhance tumor cell survival [9, 84]. For this reason, some of these studies have implied that the induction of an osteolytic environment would be appropriate during the formation of the premetastatic niche. However, it has also been noted that DTCs establish in osteogenic niches during early-stage bone colonization, before the onset of overt osteolytic metastases [85], which better reflects clinical findings.

While some preclinical studies suggest that osteolytic environments favor the implantation of DTCs, clinical evidence remains inconclusive. Few studies have assessed the relationship between bone mineral density (BMD) and metastasis risk, with most finding no association [86–88], and one reporting that low BMD may protect against bone metastasis in postmenopausal Caucasian women [89]. These inconsistencies may arise as a limitation of current preclinical models, which use specific cell lines with predefined characteristics and metastasis induction techniques that do not accurately reflect the great biological variability of human tumors in clinical contexts [90]. Additionally, most preclinical research is based on mice, which exhibit osteoimmune differences from humans that may be relevant to the Tumor-Bone Axis [91]. These factors may contribute to a more rapid transition from initial osteogenic environments to osteolytic states in commonly used preclinical models, compared to the clinical setting where this process may take several years to unfold [90].

Clinical evidence indicates that the BMD fluctuates throughout the course of breast cancer. Postmenopausal women often present with high BMD at diagnosis [92–100], a factor associated with increased disease risk and a poorer prognosis [89, 101–111]. After diagnosis, however, BMD declines faster in women with breast cancer compared with those who do not have the disease, regardless of menopausal status [112–118], and this decline can persist after treatments are completed, depending on the therapies used [119, 120]. Bone loss might be especially relevant in recurrence, considering osteolysis-derived factors may facilitate tumor survival [121, 122]. Most recurrences are metastatic—predominantly to bone—and represent the primary cause of mortality in breast cancer [123, 124]. Bone loss after treatment has been linked to earlier skeletal metastases, which antiresorptive agents may delay [87, 125, 126]. As noted, both CTIBL and bone metastasis contribute to SREs, substantially affecting the quality of life of survivors [2].

Imbalanced bone resorption and bone formation are hallmark features in breast cancer bone metastases—the most common site of distant spread [127]. These lesions are typically osteolytic, driven by increased osteoclastic activity over osteoblastic bone production [128]. As MSCs differentiate into osteoblasts or adipocytes, reduced osteoblastogenesis often leads to marrow adiposity, potentially impairing hematopoiesis [129]. Less commonly, metastasis presents as osteogenic or mixed lesions, highlighting the heterogeneity of skeletal involvement [130]. Consequently, the extent of hematopoietic disruption due to altered bone remodeling may vary substantially, even within the same patient. Given the distribution of hematopoietic tissue across multiple bones, this impact is generally negligible at the systemic level, although its local effects within the tumor-bone microenvironment warrant further investigation. A summary of the clinical scenarios associated with metabolic imbalances of bone in breast cancer can be seen in Table 1. Since these alterations are common and often present at the time of diagnosis, it is necessary to investigate the interaction between breast tissue and bone before the tumor manifests.

**Table 1:** Clinical scenarios related to bone metabolic imbalances in breast cancer

Clinical scenarios	Examples	Ref.
Hematopoiesis disorders	Anemia, widening of red cell distribution, myeloid-derived immunosuppressive cells, clonal hematopoiesis of indeterminate potential, and extramedullary hematopoiesis.	[60–65]
Bone remodeling disorders	Increased bone mineral density at diagnosis, Enhanced bone mineral density loss.	[92–100, 112–118].
Metastatic lesions	Osteolytic lesions, osteosclerotic lesions, mixed lesions.	[128, 130]
Skeletal related events	Bone pain, pathological fracture, hypercalcemia, spinal cord compression.	[131–141]

## Early development of the Tumor-Bone axis

Contrary to the traditional view that tumors arise from a single mutated cell undergoing clonal expansion, breast cancer is primarily driven by a small population of breast cancer stem cells (BCSCs). These cells—constituting only 0.1.1% of the primary tumor—are key mediators of tumorigenesis, metastasis, therapy resistance, and recurrence [4]. Additionally, the malignant transformation of surrounding cells is also crucial for the development of tumors. The breast tumor microenvironment (TME) includes several cell types with altered phenotypes, such as cancer-associated adipocytes and cancer-associated fibroblasts, which maintain constant crosstalk with cancer cells [142, 143]. Other key contributors to early tumorigenesis, including tumor-associated endothelial cells, tumor-associated platelets, and immune cells, are recruited from the bone marrow [144].

**Fig. 2:**
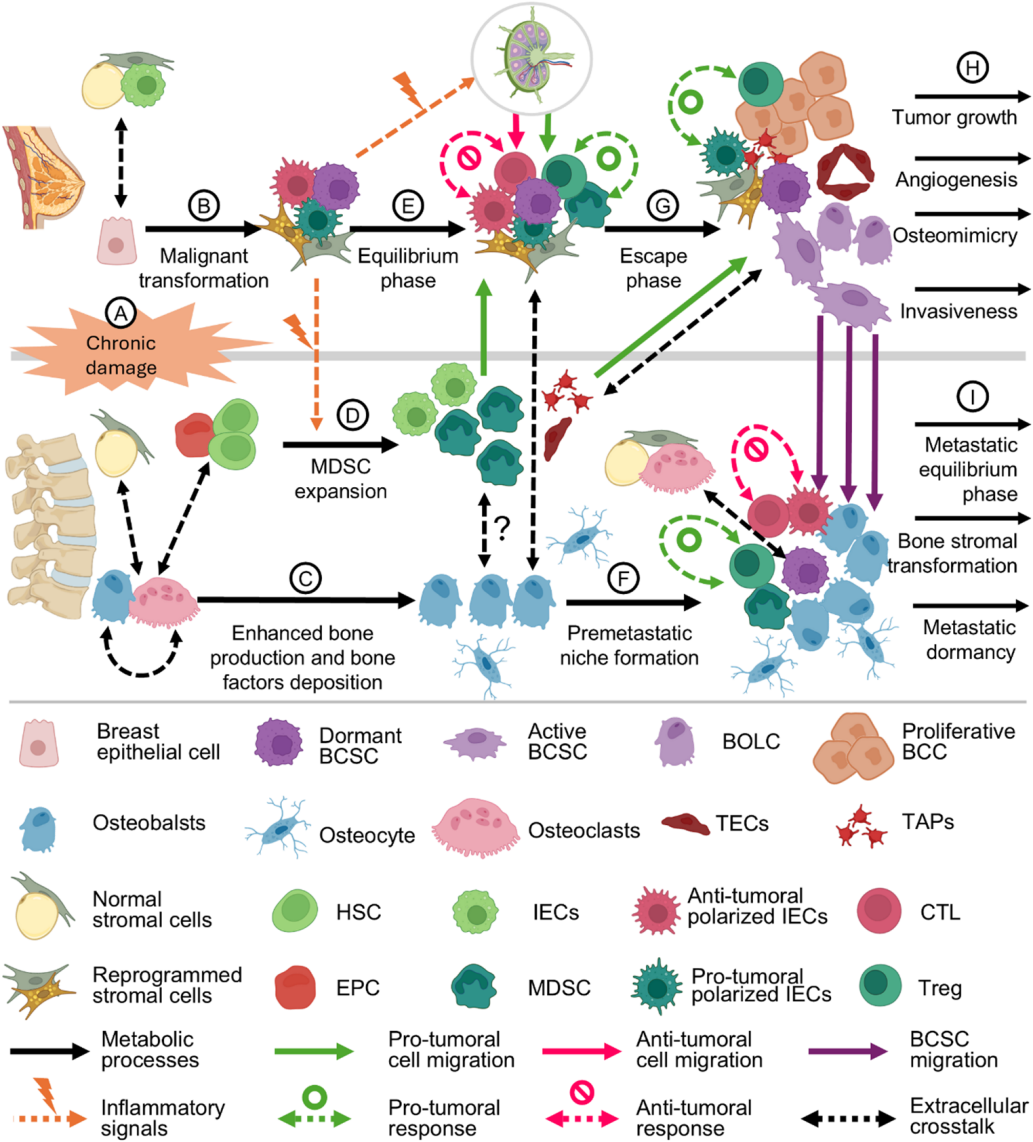
Early imbalances leading to tumorigenesis result in the formation of the Tumor-Bone Axis. (**A**) Chronic stressors triggers cooperative crosstalk among breast-tissue resident cells. (**B**) This crosstalk leads to malignant transformation and metabolic reprogramming. (**C**) In parallel, these stressors also impact the crosstalk between bone-resident cells, potentially enhancing bone formation and bone matrix-factors deposition. (**D**) After malignant transformation, an inflammatory response promotes expansion of pro-tumoral MDSCs in the bone-marrow and their recruitment. (**E**) Other recruited cells include bone-marrow derived IECs, which polarize upon arrival, and lymph-nodes derived CTL and Tregs, engaging in respective anti-tumoral and pro-tumoral immune response during the equilibrium phase. (**F**) Breast-bone communication induce premetastatic niche formation with a potential role of MDSCs expansion prior their migration. (**G**) Predominant pro-tumoral response during escape phase promote angiogenesis and activation of BCSCs. (**H**) After this, several oncological processes are enabled to facilitate early waves of dissemination to the bone. (**I**) A metastatic equilibrium phase begins, during which invasive BSCS remain dormant within osteogenic niches and induces metabolic reprogramming in local stromal cells. The various extracellular signals used in all these dialogues and immune responses include extracellular vesicles. BCC, Breast Cancer Cell; BCSC, Breast Cancer Stem Cell; BOLC, Breast Osteoblast-Like Cell; CTL, Cytotoxic Lymphocyte; EPC, Endothelial Progenitor Cell; HSC, Hematopoietic Stem Cell; IEC, Innate Effector Cell; MDSC, Myeloid-Derived Suppressor Cell; TAP, Tumor-Associated Platelet; TEC, Tumor-Associated Endothelial Cell; Treg, Regulatory T Cell

Given that the generation of BCSCs and the neighboring cell transformation are crucial simultaneous steps in the development of mammary tumors, the initiation of malignancy in breast cancer aligns with the wound-oncogene-wound healing theory. This model suggests that mutations arise secondarily as an adaptive response to persistent injury, characterized by prolonged activation of oncogenes and inhibition of tumor suppressor genes [5]. It is notable that the usual damaging stimuli to breast tissue also affect bone metabolism. Thus, early bone alterations may occur in parallel with tumor initiation, potentially explaining why skeletal metabolic changes are often present at the time of diagnosis. Figure 2 schematically represents the cellular crosstalk and the alterations that occur in parallel in breast and bone tissue during the early imbalances leading to tumor development.

### Early imbalances leading to the Tumor-Bone axis establishment

Breast cancer and bone integrity share key determinants, such as lifelong estrogen exposure, increased adiposity, and phosphate imbalance (Fig. 2A). Estrogens promote epithelial proliferation in the breast and are implicated in up to 75% of breast malignancies [145]. They also stimulate osteoprotegerin production, which inhibits osteoclast activity [146]. Adipose tissue contributes to estrogen synthesis but also drives chronic inflammation and oxidative stress, which are related to both carcinogenesis and bone metabolic adaptations [147]. Phosphate metabolism, regulated by osteoblasts, can promote excessive bone mineral deposition and tumor progression when dysregulated [148]. The convergence of these stressors may facilitate early inter-tissue communication, even before malignancy develops. However, further research is needed to identify the mediators of this pre-malignant dialogue and how it changes through malignant transformation.

Chronic microenvironmental stress not only reshapes tumor-initiating cells but also affects the surrounding stromal populations. Malignant transformation may begin with metabolic reprogramming as an adaptive response to prolonged environmental insults [6]. Stromal support is essential for survival under these conditions, enabled by complex molecular exchanges within the TME [3, 149]. Mitochondrial dysfunction emerges as a hallmark of this adaptation [150, 151], collapsing mitochondrial-nucleus communication and promoting malignant traits, including atavistic-embryonic phenotypes [6, 152]. The Wnt signaling pathway is a key player in this context. Being central to mammary gland development and allowing stem cells to retain pluripotency and self-renewal, this breast atavistic pathway is commonly upregulated in breast cancer, though rarely through mutations, suggesting an epigenetic mechanism [153, 154]. Moreover, Wnt signaling drives stromal dedifferentiation, converting mature adipocytes into cancer-associated adipocytes and cancer-associated fibroblasts [155, 156]. This is also the main signaling pathway governing osteoblast differentiation [19, 157]. Therefore, chronic damage may lead to epigenetic upregulation of the Wnt pathway, potentially linking early oncogenic and osteogenic processes (Fig. 2B and C), as well as subsequent tissue remodeling and early immune surveillance interaction.

In the normal breast, resident immune cells regulate tissue remodeling. Intraepithelial lymphocytes (IELs) and tissue-resident macrophages are found within the mammary epithelium and stroma, respectively [158]. Being essential during lactogenesis, IELs increase during pregnancy and lactation, while macrophages become more active at the end of this period [158–160]. Although their immune roles in the breast are less explored, IELs are well characterized in the gut, where distinct subsets—ranging from T cells to Innate Lymphoid Cells—mediate cytotoxicity, immune tolerance, and wound healing via interactions with the microbiome and fibroblasts [161]. Similar immune-microbiome interactions in the breast may influence tumorigenesis and progression [162, 163], but whether they also contribute to changes in distant sites, such as the bone, remains unclear. Tissue-resident macrophages respond to early tissue damage by remodeling the ECM, presenting antigens, and releasing cytokines that promote immune infiltration and the expansion and recruitment of bone marrow-derived cells into the transformed microenvironment [160] (Fig. 2D). Current evidence suggests that the Tumor-Bone Axis originates at this stage; however, further studies are needed to explore the early immune crosstalk between the breast and bone under chronic stress.

### Tumor bone axis during immunoediting

Early transformed cells must evade immune surveillance to proliferate and initiate tumorigenesis. This occurs through immunoediting, a process comprising three phases [7]. In the elimination phase, the immune system actively destroys transformed cells. During the equilibrium phase, anti- and pro-tumoral responses coexist until the latter dominates, leading to the escape phase and subsequent tumor growth and clinical detectability [7]. Immunoediting strategies vary across tumor types, influenced by the microenvironment and mutational burden acquired during transformation [164], resulting in diverse immune activity.

In breast tumors, the inflammatory infiltrate has paradoxical roles: some immune cells support anti-tumoral activity, while others promote tumor growth. The anti-tumoral infiltrate comprises various immune cells that generate cytotoxic responses through the recognition of tumor antigens [165]. Among them, CD8 + T cells are the primary effectors, supported by B cells, Tfh cells, and Th1 cells. NK cells, which derive from the bone marrow, also contribute [165]. In contrast, the pro-tumoral infiltrate suppresses cytotoxicity and perpetuates wound-healing mechanisms. This includes Tregs, Th2 cells, and bone-marrow-derived MDSCs [144, 165]. Depending on the local signals they receive upon arrival, other innate effector immune cells recruited from the bone marrow exhibit high plasticity and can adopt either a type 1 (anti-tumoral) or type 2 (pro-tumoral) phenotype, such as M1 or M2 macrophages, DC1 or DC2 dendritic cells, and N1 or N2 neutrophils [165, 166] (Fig. 2E).

The factors in the TME that favor either an anti- or pro-tumoral response in breast cancer often coexist. Pro-tumoral responses develop under stromal conditions common in breast tumors, such as hypoxia or lipid enrichment [167–169], whereas anti-tumoral responses are enhanced in tumors with a high mutational burden [164]. Consequently, cytotoxic immune cell recruitment is frequently observed in aggressive breast tumors with substantial immune infiltration, while immunosuppressive cells are consistently present across many breast tumors, including those with lower immune infiltration [164]. This results in distinct immunoediting patterns corresponding to tumor molecular subtypes, although the early recruitment of bone marrow-derived cells to the TME is a consistent feature across subtypes.

Triple-negative and HER2-enriched tumors, especially those with higher histological grades, exhibit higher mutational burdens and increased neo-antigen production. This elevated antigenic load correlates with greater inflammatory infiltration compared to luminal tumors, as well as with higher expression of immune evasion proteins, such as programmed cell death ligand 1 (PD-L1) and galectin 9 [166, 170]. Expression of these proteins leads to exhaustion of the cytotoxic response, allowing the pro-tumoral response to predominate and enabling tumor escape. Immune checkpoint blockade therapies target these evasion proteins to reactivate the elimination phase [166, 170, 171]. Accordingly, a high presence of tumor-infiltrating lymphocytes predicts a better response to these treatments [165, 166]. Additionally, bone marrow-derived cells, such as NK cells, M1 macrophages, DC1 dendritic cells, and N1 neutrophils, also contribute to this response [165, 166].

In luminal tumors, typically characterized by lower mutational burdens, tumor escape usually results from a less pronounced inflammatory infiltrate, but with a predominance of pro-tumoral immune cells. The presence of immunosuppressive infiltrates has been associated with poor prognosis, specifically in hormone receptor-positive tumors [170, 172]. Tumor-associated macrophages (TAMs) in hypoxic breast TMEs generally adopt an M2 phenotype [169], which is sustained by lipid enrichment from cancer-associated adipocytes [167]. M2 TAMs secrete cytokines that recruit pro-tumoral immune cells, including Tregs, which suppress cytotoxic activity via anti-inflammatory cytokines such as IL-10 and TGF-β [14, 149]. Moreover, the transformed cells release factors that recruit MDSCs, which, once present in the TME, exhibit potent immunosuppressive activity, differentiate into M2 TAMs, and promote additional suppressor cells recruitment [173, 174]. Thus, regardless of the immunoediting trajectory, early signaling from mammary tissue to bone is essential to initiate immune cell recruitment and shape the evolving TME.

After immune escape of early transformed cells, tumor progression and metastatic dissemination can occur. Two main models explain metastasis. In the linear progression model, metastasis develops after the primary tumor has been established, whereas in the parallel progression model, cells with metastatic potential disseminate during early stages of primary tumor development [8]. Bone marrow is the most frequent site of metastasis in breast cancer, and increasing evidence supports its association with the parallel progression model, characterized by early and repeated waves of BCSC dissemination during the onset of tumor growth [175]. In such cases, to allow parallel invasion, the process of premetastatic niche formation must initiate by, or before, the final stages of the equilibrium phase (Fig. 2F). Consequently, at this point, the Tumor-Bone Axis plays a simultaneous role in forming pre-metastatic niches and later promoting tumor growth following immune escape.

### The tumor-Bone axis during premetastatic niche formation and tumor progression after immune escape

Despite the persistent adverse conditions, the primary TME provides breast cancer cells with a supportive environment that enables their growth and progression. As reviewed so far, this support arises from the cooperative interaction with stromal cells and the establishment of local immunosuppression [176]. To thrive in secondary organs, disseminated tumor cells (DTCs) must recreate this supportive context at the metastatic site. Bone, as a highly vascularized and hypoxic organ enriched with growth and dormancy factors, can support the proliferation and stemness of DTCs [90, 177]. Notably, the cellular components present in breast tumors and in bone share similarities across their different niches (Fig. 1B and C) [22, 178, 179], although in the bone, these cells require malignant reprogramming to effectively serve as stroma for invading cancer cells. Thus, immunoediting—and its three phases—in the secondary organ is a critical step for enabling the subsequent metabolic reprogramming necessary during pre-metastatic niche formation and for allowing the future invasion of DTCs [7, 179]. Like in the primary tumor, this process continues until the initial inflammatory anti-tumoral response is finally suppressed by the inflammatory pro-tumoral response, which in the bone is also mediated by Tregs, M2 TAMs, and MDSCs [7, 179].

Among these immunosuppressive cells, MDSCs may play a particularly central role in the Tumor-Bone Axis. These cells, derived from HSCs under pathological conditions, are key facilitators of cancer progression and have been associated with poor clinical outcomes [174]. Unlike Tregs, which expand in lymph nodes, and TAMs, which acquire their M2 phenotype within TME, MDSCs expand early in the bone marrow during the immunoediting of the nascent primary tumor [180] (Fig. 2D). Given their capacity to recruit both M2 TAMs and Tregs [181], their role within the bone—prior to mobilization to the primary tumor—warrants further investigation. Exploring whether MDSCs concurrently participate in pre-metastatic niche formation in the bone while acting in immunoediting the primary tumor site could clarify the mechanisms behind the parallel progression of bone metastases in breast cancer.

The effects of MDSCs on bone-resident cells prior to their mobilization into primary TME should also be examined. Immunosuppressive cells play physiological roles in bone repair, notably by inducing MSCs to differentiate into osteoblasts and promote osteogenesis [182, 183]. It is therefore crucial to determine whether the expansion of MDSCs—during early immunoediting or pre-metastatic niche formation in bone or other organs—contributes to increased bone formation. If confirmed, this would represent a fundamental metabolic difference from mice, which are commonly used in preclinical models of breast cancer metastasis. In murine models, MDSCs expand in the spleen rather than in the bone marrow [91], potentially resulting in a weaker osteogenic response in this species. This difference could help explain why bone metastasis in mice typically shows more rapid osteolysis than is observed in clinical cases, as previously discussed, although further research is needed to determine this.

In parallel, after expanding in the bone marrow and reaching the primary TME, MDSCs promote tumor progression. Their recruitment not only facilitates immune escape but also induces angiogenesis, ECM remodeling, and epithelial-mesenchymal transition (EMT) [169, 181]. Immune evasion reinforces the interdependence between cancer and tumor stroma cells, prompting further recruitment of bone marrow-derived cells (Fig. 2G). As the tumor grows, hypoxia and altered nutrient availability exacerbate pathological changes, enabling additional mutations and ECM remodeling [149]. TAMs and cancer-associated fibroblasts secrete enzymes essential for invasion and EMT, assisting cancer cell migration [169, 184]. Tumor factors abnormally activate platelets into tumor-associated platelets, which infiltrate the TME, recruit endothelial progenitor cells from the bone marrow, and help establish tumor vasculature composed of metabolically altered tumor-associated endothelial cells that support tumor survival [144, 185]. Tumor-associated platelets also cooperate with immunosuppressive cells, further promoting EMT [144].

Beyond invasion, EMT enables breast tumor cells to express bone markers before they reach circulation. Breast osteoblast-like cells (BOLCs) are found among breast tumor cells undergoing EMT and drive tumor calcification through a process similar to bone ossification [186, 187]. BOLCs express bone markers such as RANKL, a key osteoclastogenesis regulator that also stimulates M2 TAMs to recruit additional immunosuppressive cells from bone marrow [188]. This enhances local immunosuppression, promoting the expansion and migration of BCSCs, facilitating bone metastasis [189]. Further research is needed to determine the extent to which this localized bone-like environment is essential for BCSCs to acquire osteomimicry traits necessary for bone invasion. Following EMT, tumor cells can detach and enter the circulation in waves, aided by tumor-associated platelets that protect them from immune attack while circulating [144] (Fig. 2H). Osteomimicry factors, including adhesion molecules and osteogenic elements, guide DTC homing to bone [190]. Once in bone, DTCs occupy HSC niches by expressing homing factors that anchor them to osteoblasts and enter a dormant state [177] (Fig. 2I).

The correlation between higher BMD at diagnosis and greater DTC presence requires further examination. DTCs are detected in the bone marrow of 30–50% of people with early-stage breast cancer at diagnosis [191, 192], residing in osteoblasts- and osteoprogenitor-rich niches that retain osteogenic capacity before the development of overt metastasis lesions [85]. Several DTC-guiding factors also promote osteoblast proliferation and bone matrix mineralization [190], suggesting that early osteogenic disruption may contribute to the preclinical progression of disease. However, in later stages, the Tumor-Bone Axis may shift toward predominance of other metabolic environments.

## The Tumor-Bone axis enables different metabolic States

**Fig. 3:**
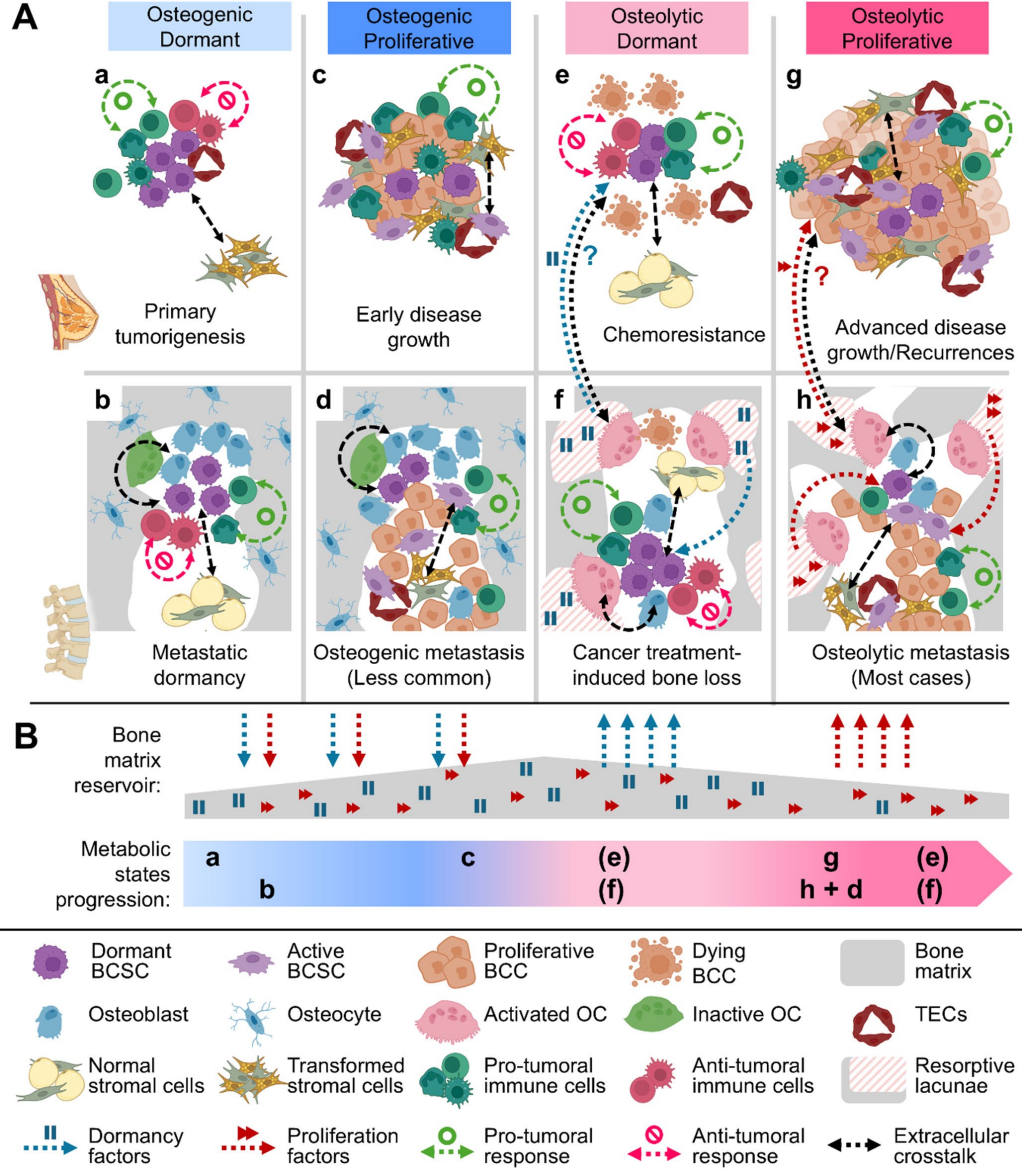
(**A**) The Tumor-Bone Axis enables four distinct metabolic states that relate to various oncological processes. Primary tumorigenesis (**a**) and initial bone micrometastases (b) are associated with a predominantly Osteogenic Dormant state. Early tumor growth (c) develops under a predominantly Osteogenic Proliferative state. Upon treatment, cancer-therapy induced bone loss (f) may lead to a predominantly Osteolytic Dormant state, potentially contributing to chemoresistance (e). Advanced tumor growth (g) and most bone metastases (h) develop within a predominantly Osteolytic Proliferative state. Some bone metastases (d) may instead present local predominance of an Osteogenic Proliferative state. Dormant states result from a balance between anti-tumoral and pro-tumoral immune responses. In all states, BCCs engage in extracellular communication with stromal cells, and in the bone, additionally interact with osteoblasts and osteoclasts. The various extracellular signals used in all these dialogues and immune responses include extracellular vesicles. The possibility of distant communication allowing the systemic use of bone-derived factors warrants further research. (**B**) The progression of predominance in the metabolic states favored by the Tumor-Bone Axis tends to shift from Osteogenic to Osteolytic states, potentially favoring increased early deposition of dormancy- and growth-related factors into the bone matrix, which may later be released during osteolytic resorption. BCC, Breast Cancer Cell; BCSC, Breast Cancer Stem Cell; OC, Osteoclasts; TEC, Tumor-Associated Endothelial Cell

By the time breast cancer becomes clinically detectable, a well-established crosstalk between the tumor and the bone has developed. This interaction can lead to distinct local outcomes in primary or secondary tumors. However, the Tumor-Bone Axis tends to favor one dominant metabolic environment at a time, reflected in clinical features associated with bone remodeling (osteogenic vs. osteolytic) and tumor cell behavior (dormant vs. proliferative). These dynamics give rise to four distinct metabolic states—Osteogenic Dormant, Osteogenic Proliferative, Osteolytic Dormant, and Osteolytic Proliferative—each with specific implications and expected signaling profiles. Their predominance shifts depending on disease progression and the timing or presence of treatment (Fig. 3).

Initially, the Tumor-Bone Axis favors an Osteogenic Dormant state. While the primary tumor transitions toward a proliferative state following immune escape (Fig. 3A-c), bone micrometastases often remain dormant under osteogenic conditions for extended periods (Fig. 3A-b). The bone marrow microenvironment—particularly pH, oxygen levels, and signals from osteoblasts and osteoprogenitors—facilitates DTC homing to endosteal niches and promotes dormancy [177]. These cues enhance their stemness, enabling DTCs to enter a quiescent state that evades immune surveillance while preserving self-renewal capacity [10, 90]. Nearby these niches, estrogen receptor expression is downregulated but restored at distal sites, a transient shift that further promotes stemness and resistance to hormonal therapies [10, 193]. Once in bone, DTCs mimic HSCs and stimulate osteoblast expansion, instructing them to maintain dormancy while hijacking signals commonly used to regulate hematopoiesis [22, 90, 194–196]. Candidate dormancy factors include bone morphogenetic protein 7 (BMP-7), angiopoietin-1 (ANG-1), stem cell factor (SCF), annexin II (ANXA-2), and growth arrest-specific 6 (GAS-6) [89]. If these dormancy signals are embedded and later released from the bone matrix, like growth factors do [31], higher BMD in earlier stages may act as a reservoir for later osteolytic metabolic states (Fig. 3B).

For secondary tumors to develop in the bone marrow, DTCs must shift from relying on dormancy signals to responding to growth factors, leading to a proliferative state. The triggers for this transition remain unclear but are thought to involve long-term crosstalk between DTCs and surrounding cells in distinct bone marrow niches [10, 194]. Only specific DTC phenotypes are prone to reactivation [197], and emerging evidence suggests that intrinsic expression of immune-evasive regulatory molecules may contribute to this process [198]. These findings imply that epigenetic reprogramming, potentially analogous to that in primary tumorigenesis, is required. One key contributor is the transformation of bone marrow adipocytes into cancer-associated fibroblasts, which promotes secondary tumor formation, chemoresistance, and immunomodulation [199, 200]. In later stages, osteoclasts play a central role in shaping the metastatic bone environment, particularly in breast cancer, where osteolytic lesions are the most common [16]. Their resorptive activity releases bone-stored growth factors that fuel tumor proliferation, thereby perpetuating the vicious cycle of bone metastasis [9, 16]. In turn, tumor cells enhance osteoclast activity through several mechanisms, including the direct secretion of RANKL, the upregulation of RANKL in osteoblasts and stromal cells, RANKL-independent pathways, and the suppression of osteoprotegerin [201]. Moreover, Tregs can express RANKL, and MDSCs may differentiate into osteoclasts in response to microenvironmental cues [201].

Building on this, the shift from reliance on dormancy signals to growth factors may depend on whether tumor cells have established a supportive stromal microenvironment, mirroring the tumor-stroma co-dependence seen in primary tumorigenesis. This model also offers a plausible explanation for the heterogeneity observed in breast cancer bone metastasis [8], as primary tumor cells continue to evolve and disseminate in successive waves, each carrying distinct genetic and phenotypic profiles. After bone-exclusive metastases, the second most common presentation of distant metastatic breast cancer is multi-organ involvement—most frequently affecting bone, liver, and brain [127]. The stemness of DTCs in bone may enable their reprogramming into phenotypes better suited for colonizing distant organs, as bone acts as a transfer station before secondary dissemination into other organs [10]. Consequent to this heterogeneity, the stromal relationships required for DTCs to thrive may vary; however, in advanced disease, the Tumor-Bone Axis typically leads to an Osteolytic Proliferative state [9, 128] (Fig. 3A-g and A-h). In rarer cases, conditions may allow bone metastatic outgrowth in association with an osteoclast-independent stroma, potentially giving rise to osteogenic metastases in a local Osteogenic Proliferative state [9, 128] (Fig. 3A-d).

Most breast cancer cases show no signs of secondary tumors at the time of diagnosis, but this does not mean that bones have not been invaded, as DTCs could be present in dormant states, as previously described. Interestingly, BMD declines after diagnosis even in patients without detectable bone metastases [112–118]. The leading cause of this decline is CTIBL, a well-studied phenomenon arising from the osteotoxicity of certain anticancer therapies [202, 203]. While the pharmacological mechanisms underlying CTIBL are beyond the scope of this work, their clinical relevance is recognized both at this stage and in later BDBC progression. Moreover, cancer treatments may alter the communication between cells involved in the Tumor-Bone Axis [204], a phenomenon that needs further characterization. Either through direct effects of CTIBL or as part of adaptive tumor response, bone loss triggers the release of bone-derived dormancy and growth factors, which DTCs may exploit to resist therapy by entering a non-replicative Osteolytic Dormant state [10, 90] (Fig. 3A-e and A-f). It remains to be determined whether these osteolysis-derived factors are also accessible to BCSCs in the primary tumor or DTCs in other organs, and if a more pronounced degradation of bone matrix increases their availability. Answering these questions is crucial, given that this chemoresistance contributes significantly to relapsing after successful treatment, the most common form of advanced disease, and the leading cause of breast cancer mortality [205].

## Extracellular vesicles as key mediators in the Tumor-Bone axis

As discussed so far, multiple types of mediators contribute to the Tumor-Bone Axis (Table 2). Among them, EVs are likely the most versatile and suitable. Their advantages include a remarkable diversity and selectivity of cargo, the ability to mediate signaling through autocrine, paracrine, or endocrine mechanisms, and—particularly in cancer—their organotropism and capacity to cross biological barriers [206]. Moreover, the expanding field of EV research has highlighted their potential not only as valuable biomarkers—with applications in liquid biopsies and prognostic assessment—but also as therapeutic tools [206]. Investigating the role of EVs in various forms of communication within the Tumor-Bone Axis and across its associated metabolic states may therefore offer critical insights into this model and facilitate its translation from bench to bedside.

**Table 2:** Types of mediators involved in the Tumor-Bone axis

Type of mediators	Examples	References
Cell migration and direct interaction	BMDC recruitment into TME, BCSC dissemination to bone	[207–209]
Adhesion molecules	CXCR4, CXCL12, VCAM 1	[90, 210, 211]
Cytokines	IFN-γ, TNF-α, IL-12, IL-4, IL-10, IL-13	[14]
Soluble proteins and bone matrix-derived factors	RANKL, OPG, ALP, MMP3, IGF-1, TGF-β, MMP3, BMP-7, ANG-1, SCF, ANXA-2, GAS-6	[9, 16, 90, 177, 188, 195]
Nutrient imbalances	Calcium, phosphate, lipids	[9, 148, 167]
Hormones	Estrogens, FGF 23, PTHrP	[9, 193, 212]
Extracellular vesicles	Oncosomes, stromal-derived EVs, immune-derived EVs, matrix vesicles, chemo-EVs	[14, 15], 213– [215]

ALP, Alkaline Phosphatase; ANG-1,Angiopoietin-1; ANXA-2, Annexin A2; BCSC, Breast Cancer Stem Cells; BMDC, Bone Marrow-Derived Cells; BMP-7, Bone Morphogenetic Protein 7; Chemo-EVs, Chemotherapy-induced Extracellular Vesicles; CXCL12, C-X-C Motif Chemokine Ligand 12; CXCR4, C-X-C Motif Chemokine Receptor 4; EVs, Extracellular Vesicles; FGF 23, Fibroblast Growth Factor 23; GAS-6, Growth Arrest-Specific 6; IFN-γ, Interferon gamma; IGF-1, Insulin-like Growth Factor 1; IL, Interleukin; MMP3, Matrix Metalloproteinase 3; OPG, Osteoprotegerin; PTHrP, Parathyroid Hormone-related Protein; RANKL, Receptor Activator of Nuclear Factor Kappa-B Ligand; SCF, Stem Cell Factor; TGF-β, Transforming Growth Factor Beta; TME – Tumor Microenvironment; TNF-α, Tumor Necrosis Factor alpha; VCAM-1, Vascular Cell Adhesion Molecule 1

EVs are key mediators in the interaction between bone cells and their environment, playing essential roles in normal bone function and remodeling. For example, MSCs and HSCs release EVs that can promote HSC stemness, expansion, and guide their multilineage differentiation, depending on their origin and cargo [216]. EVs are also involved in coordinating the activities of different bone cells and niches, including the tightly regulated balance between the functions of osteoblasts and osteoclasts [13]. During bone formation, osteoblasts release matrix vesicles—a specialized type of EV—that initiate and sustain bone matrix ossification through hydroxyapatite formation [214]. Despite their established relevance in bone metabolism, further tools and experimental models are needed to study EVs in clinical contexts. Their pathological role in bone remodeling and hematopoietic disorders, as well as their therapeutic potential, is increasingly recognized [216, 217], and must also be examined in the context of the metabolic states driven by the Tumor-Bone Axis.

Importantly, EVs also contribute to metabolic reprogramming during early disruptions that promote the establishment of the Tumor-Bone Axis. Factors such as estrogen imbalance, adiposity, inflammation, oxidative stress, and phosphate dysregulation not only damage both the breast and bone microenvironments but also alter the composition and increase the release of EVs, possibly as a compensatory response to restore homeostasis [218–220]. Among these, mitochondrial dysfunction is particularly relevant, as it is both a driver and a consequence of early malignant transformation. In adipocytes and MSCs, mitochondrial stress alters EV content and enhances the release of mitochondrial-derived components, including whole mitochondria in larger EVs [221–223]. However, the extent and implications of mitochondrial material exchange via EVs during the onset of tumorigenesis remain to be fully elucidated.

Once early malignant transformation begins, EVs play a key role in immune evasion, tumor growth, and invasiveness. Their cargo evolves across immunoediting phases, promoting the formation and recruitment of immunosuppressive cells [14]. EVs also mediate lipid exchange in the TME, where epilipid functionalization within EVs influences TAM behavior, either enhancing or suppressing their tumor-promoting inflammatory effects [167, 224]. In addition to promoting the inflammatory infiltrate, EVs are also released by immunosuppressive cells to support their immunomodulatory functions [14, 15, 149]. MSCs-derived EVs may promote invasiveness in cancer cells, which also release EVs to mediate processes, including ECM remodeling, tumor-associated platelets formation, tumor vascularization, and EMT [15, 225–227]. BOLCs produced during EMT contribute to tumor calcification through a mechanism resembling bone ossification, wherein EVs play a crucial role, initiating and sustaining the mineralization process [228, 229].

The possibility of direct EV-mediated communication between tumor-resident BOLCs and bone-resident osteoblasts warrants further investigation. Similar EV-releasing osteoblast-like cells have been identified in other tissues affected by pathological calcification [230–232]. For instance, this type of cell develops from vascular smooth muscle cells in atherosclerotic calcification, a process enhanced by the uptake of matrix vesicles from bone-resident osteoblasts. Hydroxyapatite particles within these vesicles trigger mitochondrial dysfunction in vascular smooth muscle cells, leading to inflammation, calcium imbalance, osteogenic traits, and further vesicle release [233–235]. Since breast cancer is also marked by mineral imbalance, mitochondrial dysfunction, and inflammation, it is important to explore whether osteoblast-derived matrix vesicles similarly contribute to BOLC formation and tumor calcification. If so, this would suggest that bone participates more actively—and bidirectionally—in premetastatic niche formation than currently understood.

EVs play a central role in the interplay between tumor and bone cells during the formation of the premetastatic niche and the maintenance of DTCs after bone invasion. These particles contribute to creating a more permissive environment for invasive cells by enhancing osteoblast activity, promoting fibroblast reprogramming, inducing angiogenesis, and facilitating ECM remodeling [11, 15, 236, 237]. This EV-mediated communication persists after bone invasion, regulating both osteogenesis and osteolysis through interactions between bone-resident cells and DTCs [9, 10, 16], which likely helps maintain initial DTC dormancy. In later disease stages, following the progression of metabolic states resulting from the Tumor-Bone Axis, tumor-derived EVs also promote osteoclast activity during the metastatic vicious cycle, enabling the release of bone matrix-derived factors for tumor exploitation [238]. Additionally, EVs facilitate the formation of a premetastatic niche in distant organs beyond the bone [237].

The impact of cancer treatments on EVs within the Tumor-Bone Axis needs to be further examined. Chemotherapeutic agents induce genotoxic stress, altering not only the TME but also distant tissues [239]. These changes can affect EV release and cargo [215], in some cases, enhancing pro-metastatic traits by enriching EVs with proteins that activate NF-κβ signaling in endothelial cells [204]. Given the importance of this pathway in osteoclast metabolism [240, 241], it is plausible that certain treatments promote pro-osteoclastic EV profiles, potentially contributing to Osteolytic Dormant states and facilitating tumor use of bone matrix-derived factors. EV levels rise during neoadjuvant chemotherapy and decline after surgery [242], and specific EV cargo profiles have been linked to poorer treatment response [243]. It is necessary to investigate whether genotoxic stress and osteolysis-derived factors shape these EV profiles, especially in the context of chemoresistance and relapse.

**Fig. 4:**
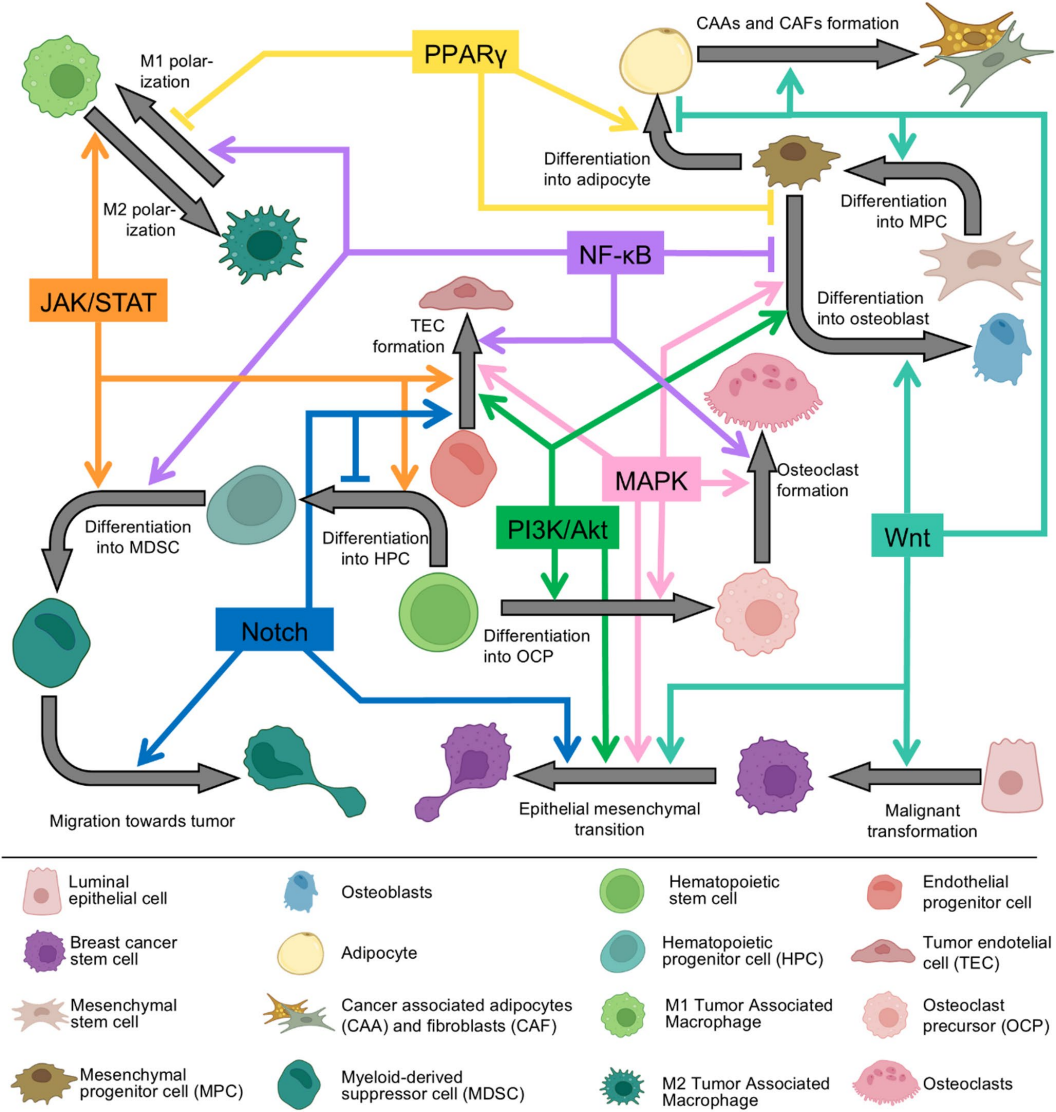
Many cell transformation and reprogramming processes derived from the Tumor-Bone Axis involve the same signaling pathways. Bold arrows (⇒) represent cellular transformation or reprogramming processes. Thin arrows (→) indicate that the connected pathway promote the signaled process. Blunt-ended lines (⊣) indicate that the connected pathway inhibits the signaled process. BCSC, breast cancer stem cell; CAA, cancer-associated adipocyte; CAF, cancer-associated fibroblast, EMT, epithelial-mesenchymal transition; EPC, endothelial progenitor cell; HPC; hematopoietic progenitor cell; HSC, hematopoietic stem cell; JAK/STAT, Janus kinase/signal transducers and activators of transcription; MAPK, mitogen-activated protein kinases; MDSC, myeloid-derived suppressor cell; MPC, mesenchymal progenitor cell; MSC, mesenchymal stem cell; NF-κB, nuclear factor kappa-light-chain-enhancer of activated B cells; PI3K/Akt, Phosphoinositide 3-kinases/Protein kinase B; PPARγ, peroxisome proliferator-activated receptor γ; OC, osteoclast; OB, osteoblast; TAM, tumor-associated macrophage; TEC, tumor-associated endothelial cell

Despite growing evidence of EV involvement in the Tumor-Bone Axis, further research is needed to clarify their mechanisms, cargoes, and clinical relevance. As discussed, key events along this axis drive distinct metabolic states through cellular reprogramming processes—such as differentiation, dedifferentiation, transdifferentiation, polarization, activation, or inactivation (Fig. 4)—which are interestingly regulated by shared signaling pathways (Table 3). These pathways can be modulated by EV cargoes, and in some cases, are also involved in EV biogenesis and release [244–246]. Consistent with the wound-oncogene-wound healing theory, they are central to refractory wound healing and are frequently dysregulated in breast cancer via epigenetic mechanisms [246, 247]. EVs carry epigenetic regulators — such as non-coding RNAs, transcription factors, and messenger RNAs— that shape the TME [248]. Thus, while many cargo types are likely exchanged among the various cells involved in this crosstalk, epigenetic regulators represent a promising focus for uncovering key mechanisms within the Tumor-Bone Axis. Particular attention should be given to the regulators of dormancy and proliferation in cancer cells, as well as to those of osteogenesis and osteolysis in osteoblasts and osteoclasts, considering the four metabolic states that result from this axis.

**Table 3:** Metabolic reprogramming processes derived from the Tumor-Bone axis and signaling pathways that modulate them

Metabolic reprogramming	Signaling pathways involved	References
Breast Cancer Stem Cell Formation and Epithelial-Mesenchymal Transition	MAPK, Notch, PI3K/Akt, Wnt	[153, 225, 249– 252]
Tumor-Associated Macrophage Polarization	JAK/STAT, NFκB, PPARγ	[167, 169, 253, 254]
Hematopoietic Stem Cell Transformation into Myeloid-Derived Suppressor Cells	JAK/STAT, NFκB, Notch	[180, 209, 255– 260]
Endothelial Progenitor Cell Transformation into Tumor Endothelial Cell and Angiogenesis	JAK/STAT, MAPK, NFκB, Notch, PI3K/Akt	[261–266]
Cancer-Associated Adipocytes and Fibroblast Formation	PPARγ, Wnt	[155, 156], 267–269]
Mesenchymal Stem Cell Differentiation into Osteoblasts	MAPK, PI3K/Akt, PPARγ, NFκB, Wnt,	[19, 267, 270– 275]
Hematopoietic Stem Cell Differentiation into Osteoclasts	MAPK, NFκB, PI3K/Akt	[201, 276–279]

J cells; PI3K/Akt, Phosphoinositide 3-kinases/Protein kinase B; PPARγ, peroxisome proliferator-activated receptor γ.AK/STAT, Janus kinase/signal transducers and activators of transcription; MAPK, mitogen-activated protein kinases; NF-κB, nuclear factor kappa-light-chain-enhancer of activated B

**Fig. 5:**
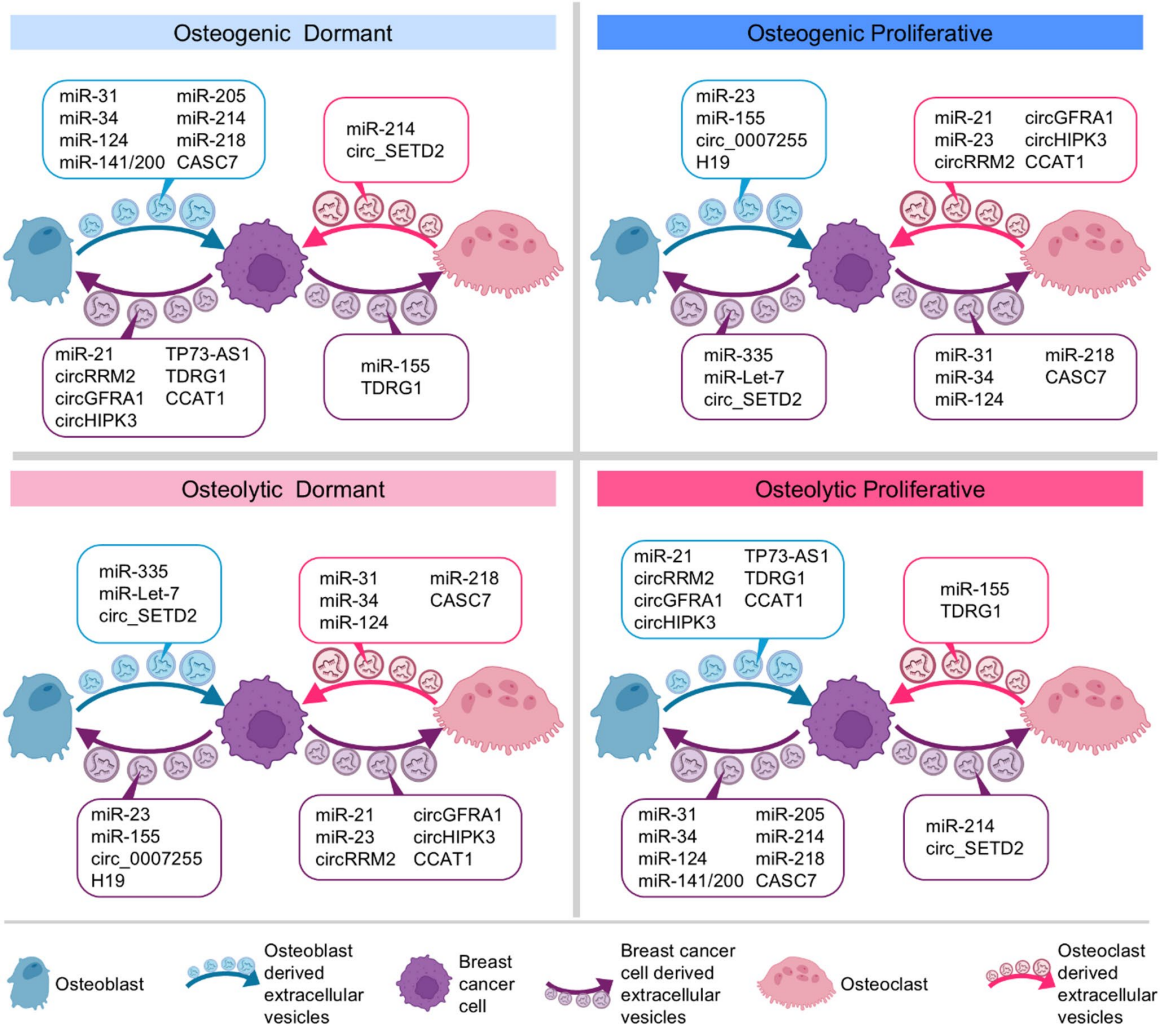
Hypothetical extracellular vesicular traffic between breast cancer cells and bone-remodeling cells within the Tumor-Bone Axis resulting in different metabolic states. This model proposes the exchange of non-coding RNAs via extracellular vesicles, leading to their downregulation in donor cells (at the tail of the arrow) and enrichment in recipient cells (at the tip of the arrow). These expression changes may contribute to the deregulation of signaling pathways, thereby altering bone remodeling and tumor activity. Further experimental validation is required to confirm these mechanisms in clinical settings

Among the epigenetic regulators found in EVs, non-coding RNAs stand out for their ability to modulate multiple signaling pathways. MicroRNAs (miRNAs), for example, inhibit gene expression by binding to messenger RNAs involved as intermediaries, modulators or effectors in key signaling cascades [280]. Competing endogenous RNAs (ceRNAs) act as molecular sponges that sequester miRNAs and block their regulatory function [280]. Depending on their expression levels, various miRNAs can either promote or suppress breast cancer cell activity and similarly influence osteogenesis or osteolysis through their effects on osteoblasts and osteoclasts [281, 282]. Several of these miRNAs—and some ceRNAs—have been identified in EVs derived from breast cancer models. Table 4 summarizes their known targets in breast and bone cells, as well as the metabolic effects of target inhibition. Considering these effects and the potential contribution of EV-mediated exchange to the downregulation of non-coding RNAs in donor cells while upregulating them in recipients, extracellular vesicular traffic for the four metabolic states of the Tumor-Bone Axis can be hypothesized (Fig. 5). Still, these models require further experimental validation, which will hopefully be possible with technological advances that are actively being developed in the field of EVs [206].

**Table 4:** Non-Coding RNAs identified within extracellular vesicles derived from breast cancer models having dual roles in cancer activity and bone remodeling

ncRNAs identified in BCEVs				Effect of miRNA Upregulation in BCCs				Effect of miRNA Upregulation in Bone Cells				
miRNA	Ref.	Inhibitory ceRNA	Ref.	miRNA target	Related pathways	Metabolic effect	Ref.	Bone cell	miRNA target	Related pathways	Metabolic effect	Ref.
miR-21	[283]	CASC7	[284]	TPM1	PI3K, MAPK	Proliferation	[285]	OBs OCs	Spry1 PDCD4	MAPK MAPK	Osteogenesis↑ Osteolysis↑	[286] [287]
miR-23	[288]	–	–	CDH1	Wnt, PI3K, MAPK	Proliferation	[289]	OBs OCs	RUNX2 PTEN	MAPK, Wnt PI3K	Osteogenesis↓ Osteolysis↑	[290] [291]
miR-31	[292]	circRRM2	[293]	WAVE3	PI3K	Dormancy	[294]	OBs OCs	STAB2 RHO A	MAPK, Wnt PI3K, MAPK	Osteogenesis↓ Osteolysis↓	[295] [296]
miR-34	[297]	circGRAF1	[298]	SNAI1	Wnt	Dormancy	[299]	OBs OCs	STAB2 TGIF2	MAPK, Wnt TGF-β	Osteogenesis↓ Osteolysis↓	[300] [301]
miR-124	[302]	circHIPK3	[302]	SNAI2	Wnt	Dormancy	[303]	OBs OCs	Dlx5 NFATc1	Wnt MAPK, NF-κB	Osteogenesis↓ Osteolysis↓	[304] [305]
miR-155	[306]	Circ_SETD2	[307]	RHOA	MAPK, PI3K	Proliferation	[308]	OBs OCs	PTEN MITF, SOCS1	PI3K MAPK, NF-κB	Osteogenesis↓ Osteolysis↓	[309] [310]
miR-141/200	[311]	TP73-AS1	[312]	WAVE3	PI3K	Dormancy	[313]	OBs	Dlx5	Wnt	Osteogenesis↓	[314]
miR-205	[315]	–	–	ZEB1	Wnt	Dormancy	[316]	OBs	RUNX2	MAPK, Wnt	Osteogenesis↓	[317]
miR-214	[318]	TDRG1	[319]	Survivine	PI3K, Wnt	Dormancy	[320]	OBs OCs	Osx, ATF4 PTEN	MAPK, Wnt PI3K	Osteogenesis↓ Osteolysis↑	[321] [322]
miR-218	[323]	CCAT1	[324]	HoxB3	Wnt	Dormancy	[325]	OBs OCs	Col1a1 TNFR1	MAPK, Wnt NF-κB	Osteogenesis↓ Osteolysis↓	[326] [327]
miR-335	[328]	circ_0007255	[329]	TNC, SOX4	Wnt	Dormancy	[330]	OBs	DKK1	Wnt	Osteogenesis↑	[331]
miR-Let-7	[332]	H19	[333, 334]	H-RAS, HMGA2	MAPK, Wnt	Dormancy	[335]	OBs	Axin2	Wnt	Osteogenesis↑	[336]

ATF4. Activating Transcription Factor 4; BCCs. Breast Cancer Cells; BCEVs. Breast Cancer-derived Extracellular Vesicles; CDH1. Cadherin 1 (E-cadherin); ceRNA Competing Endogenous RNA; Col1a1, Collagen Type I Alpha 1 Chain; DKK1, Dickkopf WNT Signaling Pathway Inhibitor 1; Dlx5, Distal-less Homeobox 5; HMGA2, High Mobility Group AT-Hook 2; HoxB3, Homeobox B3; H-RAS, Harvey Rat Sarcoma Viral Oncogene Homolog; MAPK, Mitogen-Activated Protein Kinase; miRNA, MicroRNA; MITF, Microphthalmia-Associated Transcription Factor; ncRNA, Non-coding RNA; NF-κB. Nuclear Factor kappa-light-chain-enhancer of Activated B Cells; NFATc1, Nuclear Factor of Activated T-cells 1; OBs, Osteoblast; OCs, Osteoclasts; Osx, Osterix; PDCD4, Programmed Cell Death 4; PI3K, Phosphoinositide 3-Kinase; PTEN, Phosphatase and Tensin Homolog; RHO A, Ras Homolog Family Member A; RUNX2, Runt-related Transcription Factor 2; SNAI, Snail Family Transcriptional Repressor; SOCS1, Suppressor of Cytokine Signaling 1; SOX4, SRY-box Transcription Factor 4; Spry1, Sprouty RTK Signaling Antagonist 1; STAB2, Stabilin-2; TGF-β, Transforming Growth Factor Beta; TGIF2, TGFB-Induced Factor Homeobox 2; TNC, Tenascin C; TNFR1, Tumor Necrosis Factor Receptor 1; TPM1, Tropomyosin 1; WAVE3, WASP Family Verprolin-Homologous Protein 3; Wnt, Wingless/Integrated Signaling Pathway; ZEB1, Zinc Finger E-box Binding Homeobox 1.

## Conclusions and future perspectives

As summarized in this review, extracellular communication is essential for both cancer development and bone metabolism. In breast cancer, the Tumor-Bone Axis begins at early stages and persists throughout the disease progression, facilitating events such as immunosuppressive cell recruitment, dormancy, and metastatic outgrowth. This communication involves multiple mediators, including cell contact, migration, soluble factors, nutrients, hormones, and, especially, extracellular vesicles. The Tumor-Bone Axis enables distinct metabolic states depending on whether breast cancer cells are predominantly in a dormant or proliferative state, and whether bone remodeling predominantly results in osteogenesis or osteolysis. Defining the specific roles and timing of each mediator, considering the governing metabolic states, may lead to new diagnostic and therapeutic opportunities for bone disorders in breast cancer and breast cancer in general.

Notably, several knowledge gaps remain. For instance, the influence of menopausal status and age—both independent risk factors for bone disorders— warrants attention in the context of the Tumor-Bone Axis, particularly considering that pre-diagnosis fluctuations in bone mineral density are more relevant in postmenopausal women. Likewise, the extent to which antineoplastic treatments reshape this axis, potentially contributing to chemoresistance and metastasis, needs further investigation. Differences in Tumor-Bone Axis dynamics among other osteotropic cancers, such as prostate or lung, should also be explored. Importantly, since evidence suggests that bone dissemination may occur early in breast cancer, prioritizing studies on the initial stages of the Tumor-Bone Axis could yield insights into metastasis onset. However, because the disease is not yet detectable, these early events remain difficult to study in clinical settings, yielding the need for validated surrogates for these communications. Furthermore, the search for these surrogates requires the development of more appropriate preclinical models to study cases of dormant osteogenic interactions prior to overt osteolytic ones.

Extracellular vesicles have emerged as central players in nearly every stage of the Tumor-Bone Axis. Advances in extracellular vesicle research may therefore help clarify early communication events and lead to more effective interventions. Still, challenges remain, including the need to track them in vivo, understand their organotropism, and identify specific environmental triggers for their release. Despite these obstacles, progress in the field continues to accelerate, offering hope for refined research strategies to study the Tumor-Bone Axis, potentially leading to improved diagnosis, treatment, and ultimately, better outcomes for patients with breast cancer-associated bone disorders.

## Data Availability

No datasets were generated or analysed during the current study.
